# Terazosin Activates or Inhibits Human Phosphoglycerate Kinase 1 on a Dose-Dependent Basis: Mechanistic and Structural Insights by NMR

**DOI:** 10.1021/jacsau.6c00823

**Published:** 2026-09-24

**Authors:** Matthias Bütikofer, Felix Torres, Dhiman Ghosh, Nils Lorz, Roland Riek, Julien Orts

**Affiliations:** † Institute for Molecular Physical Science, Vladimir Prelog Weg 2, 8093 Zürich, Switzerland; ‡ NexMR AG, Wiesenstrasse 10A, 8952 Schlieren, Switzerland; § Department of Pharmaceutical Sciences, University of Vienna, Josef-Holaubek-Platz 2, A-1090 Vienna, Austria

**Keywords:** Phosphoglycerate kinase 1 (PGK1), Terazosin, NMR spectroscopy, Chemical shift perturbation, Protein−ligand binding, Warburg effect, Parkinson’s disease

## Abstract

Phosphoglycerate kinase 1 (PGK1) is a key metabolic enzyme responsible for ATP production, and its dysregulation is linked to the Warburg effect in cancer and to neurodegenerative diseases such as Parkinson’s. Recent studies indicate that terazosin, an FDA-approved drug, enhances PGK1-mediated ATP production. However, X-ray crystallography indicates that terazosin binds at a site that partially overlaps the nucleotide-binding site, thereby inhibiting PGK1. In this study, we employed solution-state nuclear magnetic resonance (NMR) chemical shift titrations to confirm that terazosin binds at a site that partially overlaps the nucleotide-binding pocket of PGK1, stabilizing the enzyme in its open conformation, which is accessible to substrates and products. This binding shifts the equilibrium toward the open state, enhancing product release at low concentrations while blocking substrate binding at higher concentrations. These findings are strengthened by the 3D NMR^2^ structure determination of the PGK1-terazosin complex, using selective unlabeling combined with subsequent partial amino acid assignments, which validates the available X-ray structure.

## Graphical abstract

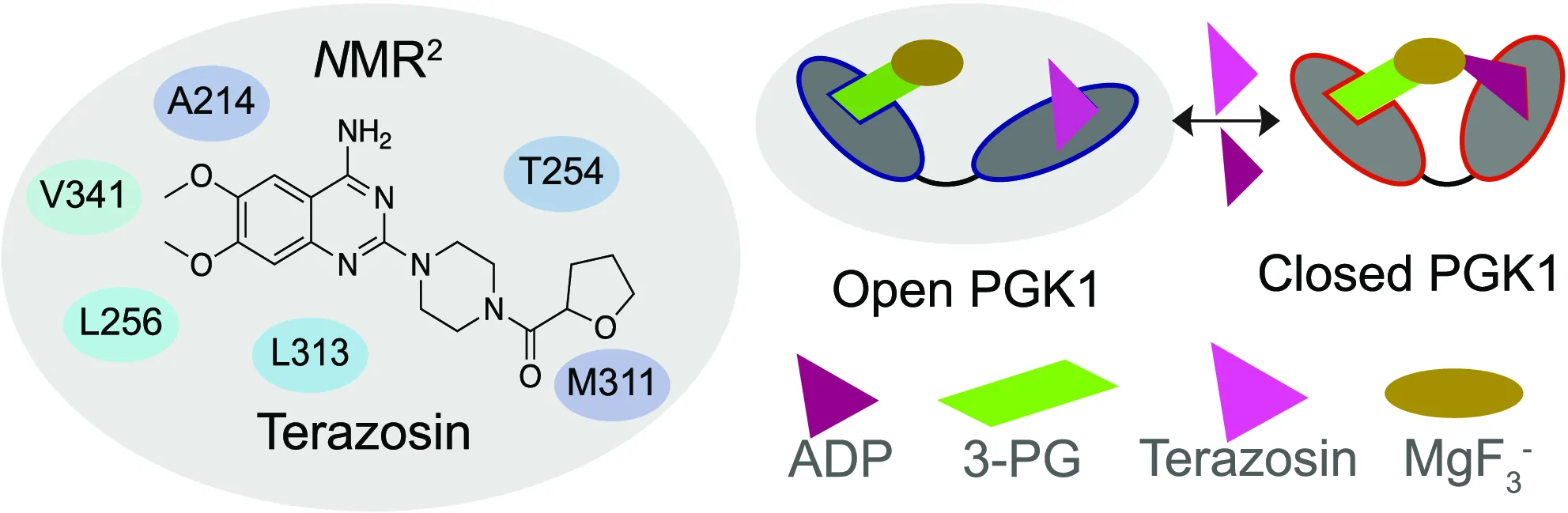

## Introduction

Phosphoglycerate kinase 1 (PGK1) catalyzes a key step in glycolysis, facilitating the magnesium-dependent transfer of a phosphate from 1,3-bisphosphoglycerate (BPG) to ADP, yielding ATP and 3-phosphoglycerate (3PG).^1^ Structurally, PGK1 consists of two domains linked by an α-helix: the N-terminal domain binds BPG/3PG, while the C-terminal domain binds ADP/ATP. X-ray crystallographic studies have shown that PGK1 adopts an open, substrate-accessible, thermodynamically favored conformation and a closed conformation required for phosphate transfer.^2,3^

The closed conformation has been captured using several strategies, including ADP and 3PG alone, AMP-PCP, AlF_4_^–^, and BeF_3_^–^ as alternative phosphoryl-transfer mimics,^4^ as well as magnesium trifluoride (MgF_3_^–^), which mimics a transition state analogue complex of 3PG-MgF_3_^–^-ADP.^5,6^

Due to its involvement in aerobic glycolysis (Warburg effect),^7^ PGK1 is potentially an attractive target for novel cancer therapies.^8,9^ Furthermore, upregulation of PGK1 and the associated increase in ATP production have been shown to protect neurons from degeneration, particularly in Parkinson’s disease.^10–12^

Previous studies showed that terazosin, an FDA-approved drug used to treat benign prostatic hyperplasia and hypertension, binds to PGK1 and is associated with increased cellular ATP levels. This increase has been proposed to enhance the activity of ATP-dependent processes, including the Hsp90 chaperone. Such effects have been linked to cytoprotective effects under stress conditions and to beneficial outcomes in models of Parkinson’s disease.^13–15^

However, in the reported X-ray structure of the complex, terazosin binds in the nucleotide binding domain with partial overlap of the ADP binding site, suggesting direct competition. Despite this, terazosin enhances ATP production at low concentrations and acts as an inhibitor at higher concentrations.^13^ Although a mechanism involving enhanced product release was proposed in this earlier work and was also supported by a recent study using a mass action model,^16^ the experimental validation has been lacking, despite the development of terazosin analogs for neuroprotection.^17^ The present work confirms the previously proposed models that hypothesized a unique binding site for terazosin, despite dose-dependent bidirectional modulation (activation at low and inhibition at high concentration) of PGK1.

Prior NMR studies on PGK1 have achieved backbone assignments and investigated the catalytic cycle, revealing the formation of the transition-state analog (TSA) complex, which can be readily distinguished by significant chemical shift changes.^18,19^

Here, we use chemical shift perturbation analysis and solution NMR structure calculation using NMR^2^,^20^ to confirm that terazosin and ADP occupy partially overlapping binding sites on PGK1. NMR^2^ is a method that determines the three-dimensional structure of a small-molecule ligand bound to a protein from intermolecular NOE-derived distance restraints alone, without requiring full resonance assignment of the protein. ^15^N titration data indicate that terazosin stabilizes the open PGK1 conformation, consistent with a mechanism in which terazosin facilitates product release at low concentration. The NOE data used for structure determination provide no evidence for a distinct alternative binding pocket under solution conditions, and the NMR^2^ structure calculation converges on the X-ray structure.

## Results and Discussion

### ^1^H–^15^N HSQC-Based Titrations Reveal That Terazosin Stabilizes the Open Conformation of PGK1

To test the hypothesis proposed by Chen et al.^13^ and Riley. et al.^16^ that terazosin enhances PGK1-mediated ATP production by promoting product release, we conducted a series of [^15^N, ^1^H]-HSQC titration series with ADP and terazosin. For simplification, we refer to all [^15^N,^1^H] backbone correlation experiments collectively as HSQC-type titrations throughout the text, even if TROSY experiments were measured, details given in Materials and Methods.

Figure 1A displays HSQC spectra of PGK1 in its open (blue) and closed (red) conformations, recorded in the apo state or in the presence of excess 3PG, MgF_3_^–^, and 2 mM ADP. MgF_3_^–^ was formed in situ by adding MgCl_2_ and NH_4_F to the sample buffer directly, enabling the study of the transition-state analog (TSA) complex.^21^ Partial backbone assignment for the closed form of hPGK1 is available (BMRB 27,022),^22^ while the open state remains unassigned. By using the titrations conducted in this study, a partial assignment of the open state was achieved.

**Figure 1:**
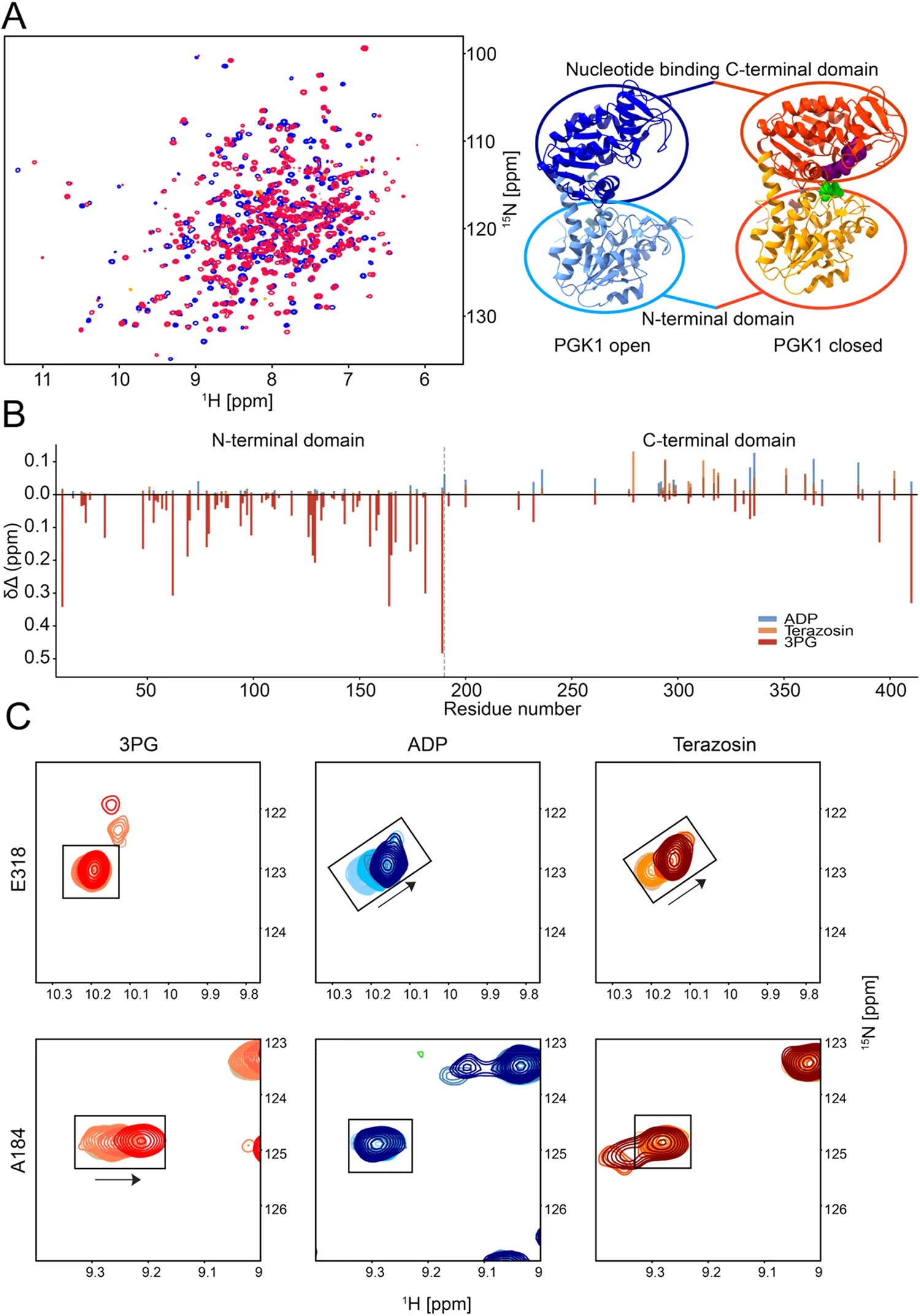
^1^H-^15^N HSQC-based titrations reveal terazosin CSPs mostly influence on the C-terminal domain of hPGK1. (A) Overlay of [^1^H,^15^N]-HSQC spectra of PGK1 in the open (light blue: N-terminal domain; blue: C-terminal domain) and closed (orange: N-terminal domain; red: C-terminal domain) states, illustrating the distinct spectral fingerprints of the two conformations. Right: structural representation of the open (PDB 2XE7) and closed (PDB 2WZB) states, colored according to the same scheme, with the N-terminal (3PG-binding) and C-terminal (nucleotide-binding) domains indicated. (B) Chemical shift perturbations upon titration of ADP (blue, 2 mM, 10 mM MgCl_2_), terazosin (orange, 1 mM), or 3PG (red, 10 mM) into apo PGK1, plotted against residue number. 3PG-induced perturbations are plotted in the downward direction purely for visual clarity; all values represent CSP magnitudes (eq 3) and are strictly positive. The dashed vertical line indicates the approximate domain boundary. (C) Snapshots of the ^1^H–^15^N HSQC titrations of 3PG (0, 100, 250, 10000 μM; tan to red), ADP (0, 200, 800, 2000 μM, with 10 mM MgCl_2_, light blue to navy), and terazosin (0, 25, 100, 1000 μM, tan to maroon), of the residues E318 (C-terminal domain) and A184 (N-terminal domain). More examples are shown in Figure S2.

Figure 1B shows the chemical shift perturbation (CSP) map of the titration of ADP (with MgCl_2_), terazosin (without MgCl_2_) and 3PG to apo PGK1 (A less condensed version of the CSP maps is presented in Figure S4A,B). The complete spectra of the ADP and terazosin titrations are shown in Figure S1A,B.

As expected and previously reported,^18^ the titration of 3PG (red bars, negative *y*-axis) induces the strongest changes in N-terminal domain, but also shows allosteric effects in the C-terminal domain. Figures 1C and S2C show snapshots of the 3PG titration spectra, illustrating the strong effects on A184, S61, L77, E126, E128 and L188. On the other hand, titrations of ADP and terazosin (light blue and orange bars, positive *y*-axis) show predominantly effects in the nucleotide binding domain, consistent with previous findings.^13^ Figures 1C, S2A,B show the effect on the residues E293, E318, A326 and G350. All these residues show CSP in the fast exchange regime, but it has to be noted that some residues also show intermediate or slow exchange effects but could not be tracked. The binding affinity using peak E318 for ADP and terazosin was measured to be 105 ± 25 μM and 13 ± 7 μM, respectively, aligning with previously reported values (Figure S3).^3,13^

To further test the terazosin binding, a terazosin titration in the presence of 3PG and 3PG-MgF_3_^–^ was conducted. The results are displayed in Figure S4C. As visible in the two graphs, only minor changes are visible within the titrations, showcasing that in contrast to the ADP binding, 3PG shows no counter-ligand modulation on the terazosin binding.

To examine the conformational transition to the closed state, we performed a [^15^N, ^1^H]-HSQC titration of ADP to PGK1 in the presence of MgF_3_^–^ and 3PG. A clear transition in the HSQC spectral pattern was observed, associated with domain closure (Figure 1A), as reported in previous studies.^18^ Figure 2A (blue) shows an intensity map of the HSQC PGK1 spectra in the closed state in the presence of 100 μM ADP. The intensities were double normalized, first internally against residue 292, and then against the completely closed state in the presence of 2 mM ADP. Residue 292 did barely change position or intensity during the titration, making it an ideal internal reference. Figures 2B, S5A–C support the intensity map with spectral snapshots (residues E318, A184, D67, S173, I210, N225, I235, F165, I367, T384, G394, G409, and D314), of the titration containing either 0, 200, or 2000 μM ADP. From the intensity drop in the graph and spectra with less ADP compared to the 2 mM baseline, it is visible that many peaks in the C-terminal domain move in a slow exchange regime, but also intensity drop in the N-terminal domain suggests domain movement toward the open state with less ADP. The apparent equilibrium constants associated with this conformational change were 75 ± 30 μM for A184 and 164 ± 50 μM for E318, values consistent with the ADP dissociation constant and supporting the coupling between ligand binding and domain closure. (Figure S6A)

**Figure 2:**
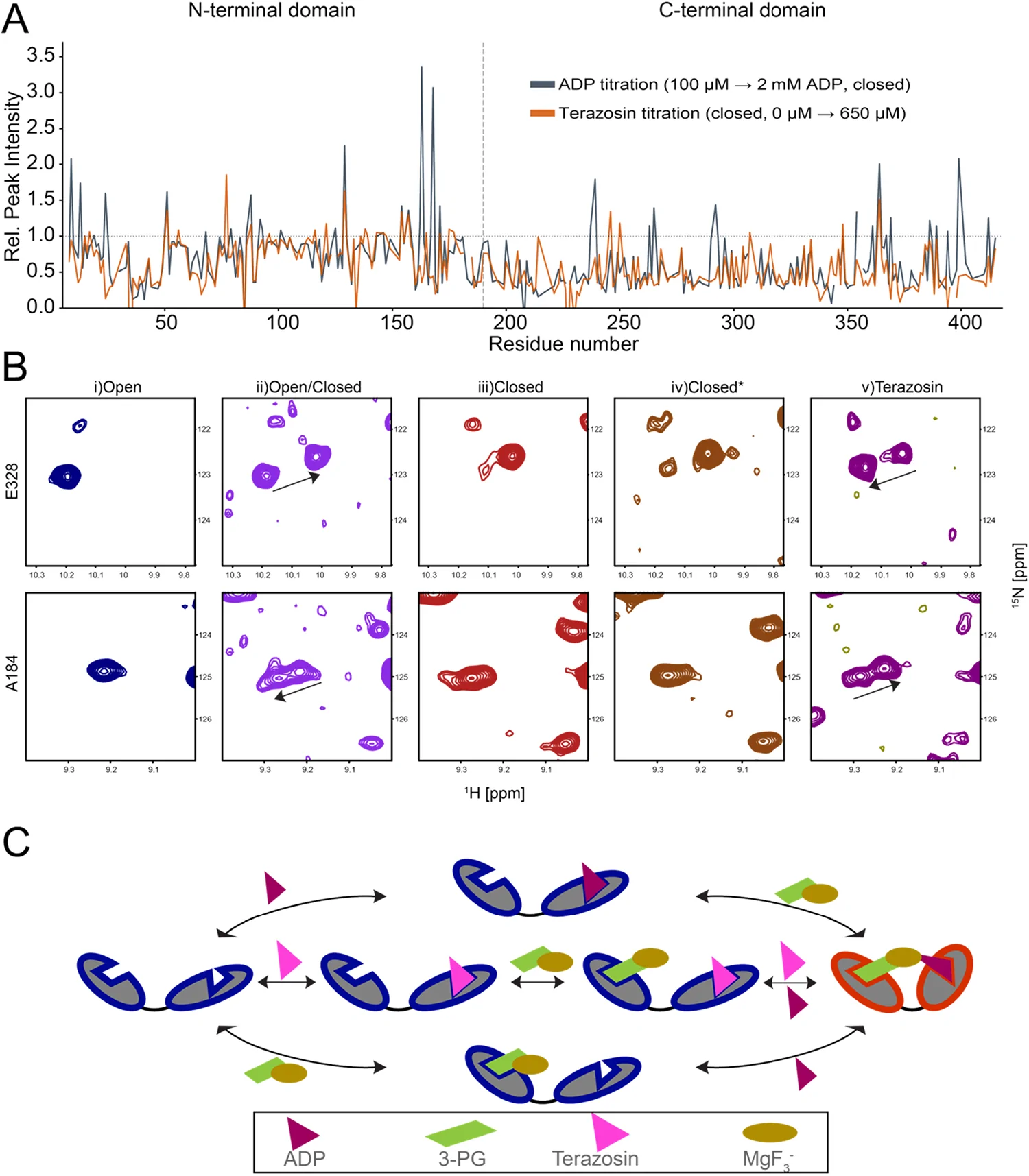
^1^H-^15^N HSQC-based titrations reveal terazosin effect on the catalytic cycle of hPGK1. (A) Relative peak intensity, tracked at chemical shift positions assigned in the closed state, across two independent titrations: ADP titrated into PGK1 in complex with 10 mM 3PG/MgF_3_^–^ (gray; 100 μM to 2 mM ADP, closed), and terazosin titrated into closed PGK1 (orange; 0 μM to 650 μM terazosin). Intensities were normalized in two steps: first, within each titration series, all spectra were internally normalized to a reference residue whose intensity and chemical shift position remained constant throughout that titration (residue 292 for the ADP series; residue 135 for the terazosin series), second, the resulting values were normalized to each residue’s own intensity in the respective fully closed reference state (2 mM ADP or 0 μM terazosin, dotted line, relative intensity = 1.0). Note that the two titrations were performed under different background ADP concentrations (2 mM and 1 mM, respectively). (B) Snapshots of the ^1^H–^15^N HSQC titrations of the closing with ADP (i) 10 mM 3PG, 10 mM MgF_3_^–^, (ii) + 200 μM ADP and (iii) +2 mM ADP and opening with terazosin (iv) + 1 mM ADP, 0 μM terazosin, (v) + 1 mM ADP, 650 μM terazosin of the residues E318 (C-terminal domain) and A184 (N-terminal domain). More examples are shown in Figure S5. (C) Proposed mechanistic scheme for ligand-induced domain closure and reopening of PGK1 with terazosin.

Finally, titrating terazosin into the closed form of PGK1 (preincubated with MgF_3_^–^ and 3PG, 1 mM ADP) was conducted, and similarly to the ADP titration, the intensities of the closed peak assignment were followed during the titration (Figure 2A, orange). The titration step at 650 μM terazosin and 1 mM ADP was normalized with the internal residue 135 and against the baseline without terazosin. Again, a significant drop in the nucleotide binding domain, but as well a drop in the N-terminal domain is visible. Furthermore, Figures 2B, S5D,E show snapshots of the spectra, in which it is visible that the peaks which appeared or disappeared during the ADP titration show the opposite effect now, indicating that a reversed transition is induced by terazosin addition. Additionally, the apparent opening equilibrium constants are 17 ± 33 μM (A184) and 38 ± 36 μM (E318), values comparable to terazosin binding to the open state (Figure S6B).

The findings that (i) terazosin binds into the nucleotide binding domain and induces little effects in the N-terminal domain upon binding, (ii) 3PG-MgF_3_^–^ has no major effect on terazosin binding and, (iii) terazosin shifts the equilibrium back into the chemical shift pattern of the open state, support the idea of a mechanism in which terazosin facilitates product release by stabilizing the open conformation at low concentrations, while acting as a competitive inhibitor at higher concentrations.

To further validate this mechanism, we next determined the solution structure of the PGK1-terazosin using the NMR^2^ approach.

### NMR^2^ Structure of Terazosin in Complex with hPGK1

To elucidate the structural basis of terazosin binding and validate the proposed product release mechanism, we determined the 3D structure of the hPGK1–terazosin complex using the NMR^2^ approach,^23^ following our established protocol adapted for this large system.

We recorded a series of 2D F_1_-[^13^C,^15^N]-filtered ^1^H–^1^H NOESY experiments with mixing times of 20, 60, 80, 100, and 120 ms on ^13^C, ^15^N-labeled hPGK1 in complex with unlabeled terazosin (Figure 3A shows a segment of the spectrum at 100 ms mixing time). These experiments enabled us to assign terazosin resonances and extract intra- and intermolecular distance restraints via NOE cross peaks between terazosin and the unassigned methyl groups of PGK1. Cross peaks were selected for restraint generation only when they could be unambiguously assigned to a specific ligand proton and showed no overlap with protein–protein NOEs in the corresponding unfiltered spectrum. Figures S7 and S8 show the NOE build-up curves obtained from the NOE cross peaks. The extracted intermolecular distances are summarized in Tables S1 and S2, providing a distance network between terazosin and the relevant PGK1 methyl groups for NMR^2^ structure calculations (Figure 3B).

**Figure 3:**
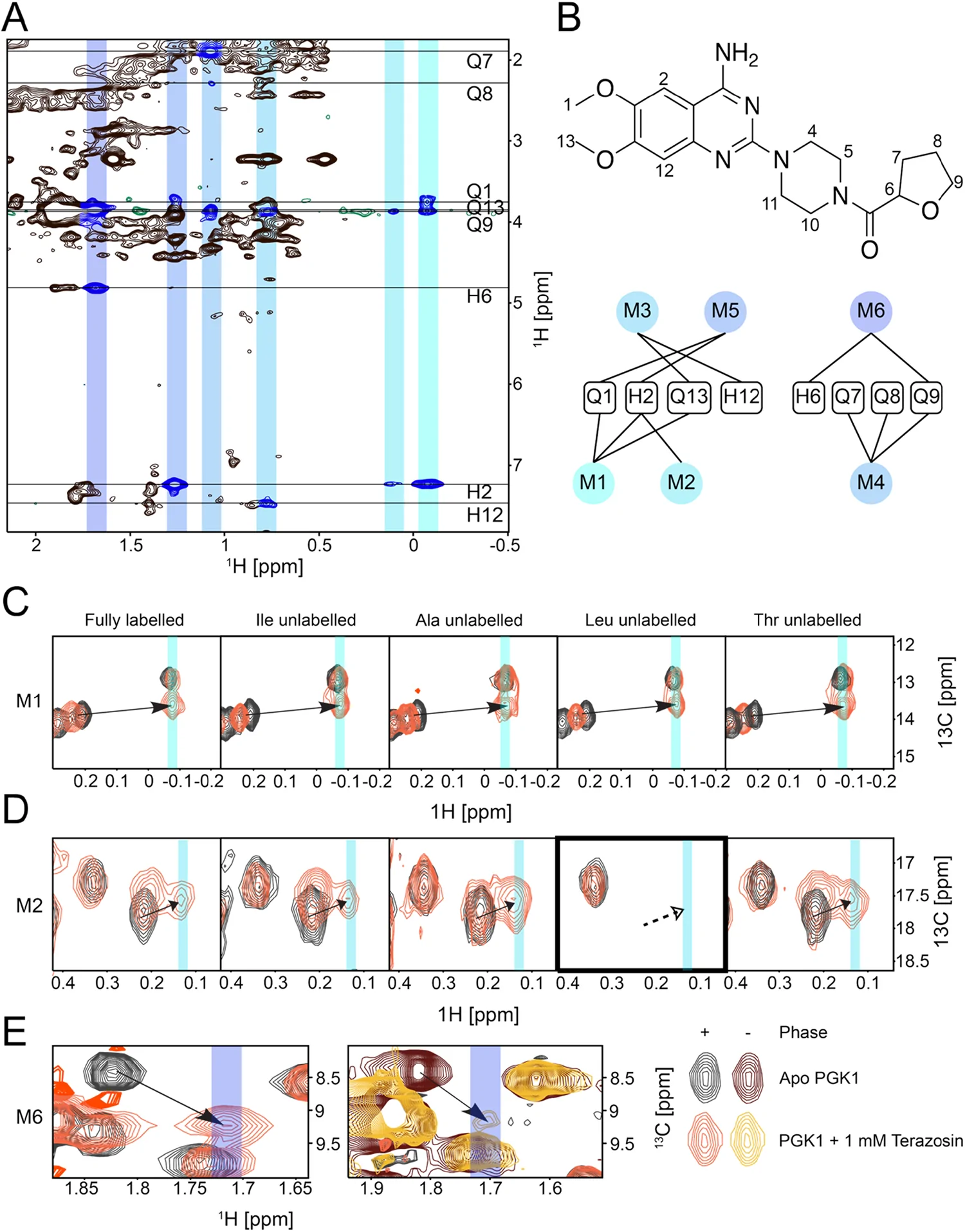
Intermolecular NOEs and selective unlabeling enable NMR^**2**^ structure calculation of the hPGK1–terazosin complex. (A) Segment of the F_1_-[^13^C,^15^N]-filtered NOESY spectrum (100 ms mixing time) showing intermolecular NOEs between terazosin and PGK1 methyl groups. (B) Intermolecular distance network derived from NOE build-up analysis, for the NMR^2^ structure calculation. Cross-peaks used are highlighted in blue. (C–E) Illustration of the selective-unlabeling strategy for partial methyl assignment. [^13^C, ^1^H]-HSQC spectra of hPGK1 selectively unlabeled in isoleucine, alanine, leucine, or threonine, recorded in the absence (black) and presence (red) of 1 mM terazosin. Arrows track the chemical shift perturbation of each methyl upon terazosin binding; colored bars correspond to the NOE cross peaks highlighted in Figure 3A. (C) M1 is detected in all unlabeled samples, identifying it as valine. (D) M2 is undetectable only in the leucine-unlabeled sample (boxed; dashed arrow marks the expected position), identifying it as leucine. (E) Standard (left) and constant-time (right) [^13^C,^1^H]-HSQC of M6; the negative phase in the constant-time spectrum (see legend) confirms its assignment as methionine. A more detailed analysis is found in SI Figure S10.

Due to the absence of intramolecular NOEs within the piperazine ring of terazosin, we could not assign this region, leaving the middle portion of terazosin without direct distance restraints. However, we observed strong unidirectional NOEs at an F_1_ resonance frequency of 3.2 ppm, potentially arising from the piperazine ring, although overlap with other resonance frequencies (e.g., M3) could not be ruled out.

Given the size of PGK1 (44.5 kDa) and the sparsity of the distance network, we implemented additional strategies to improve the convergence of the NMR^2^ calculation. We performed normal and constant time [^13^C, ^1^H]-HSQC-based titrations of terazosin into PGK1 (Figure S10), connecting the apo (black) and bound (red) methyl signals. To enable partial assignment of methyl groups, we prepared ^13^C,^15^N labeled PGK1 samples with selectively unlabeled residues (alanine, leucine, isoleucine, threonine) and recorded [^13^C, ^1^H]-HSQC of these selectively unlabeled samples (Figures S9 and S10) in the absence (black) and presence (red) of terazosin.^24^ This approach allowed a partial assignment following the logic presented in Figure 3: (C) The methyl peak correlated to the NOE cross peak is visible in all unlabeled spectra and can be assigned to a valine. If the methyl peak is missing in one of the spectra (D), it can be assigned to this amino acid. (E) A negative contour in the ct-HSQC identifies a peak as methionine. M1-M6 were subsequently assigned:

M1 was identified as a valine, since the HSQC peak is visible in all four spectra (Figure 3C). Figure 3D shows that M2 is a leucine, as its peak is missing in the leucine unlabeling variant. The HSQC cross peak for M3 could not be conclusively identified due to regional spectral crowding. M4 was assigned as alanine or threonine, based on the absence of signal in the respective spectra (Figure S10). M5 was identified as an alanine due to the disappearance of the apo peak (Figure S10). M6 was identified as methionine, based on the negative cross-peak intensities characteristic of methionine methyl groups in the ct-[^13^C, ^1^H]-HSQC spectrum (Figure 3E). All relevant partial assignments are listed in Table S2. Notably, all peaks exhibited intermediate chemical exchange behavior, leading to line broadening and, in some cases (e.g., M6), reduced detectability in the ct-HSQC.

Incorporating these partial assignments and NOE-derived distances enabled a successful NMR^2^ structure determination with a final target function of 4.51 and van der Waals violation of 0.00003. The resulting assignment based on the NMR^2^ calculation is presented in Table S3. Figure 4A shows the overlay of the water-refined NMR^2^ structure (light pink, PDB ^32^PI) and the published X-ray structure (blue, PDB 4O33). The NMR^2^ structure exhibits a slight displacement relative to the X-ray structure, with the quinazoline and the piperazine rings adopting similar conformations, while the furan ring shows a slight tilt.

**Figure 4:**
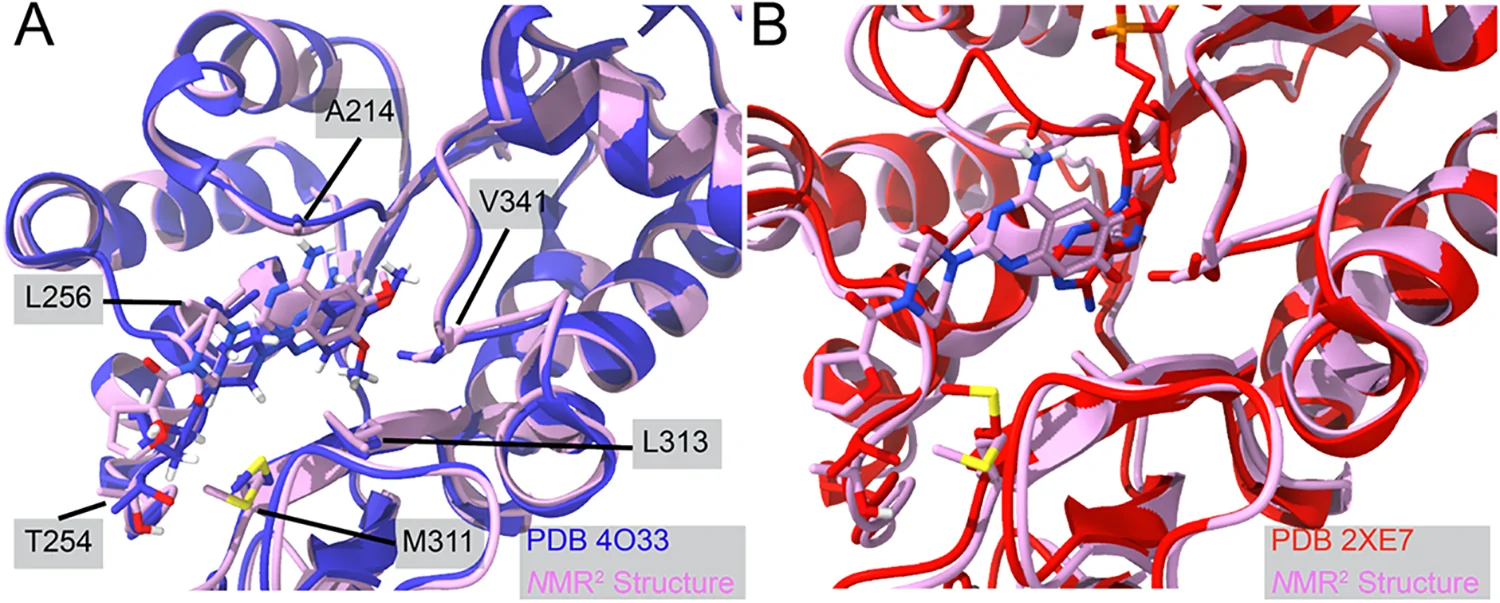
Structural comparison of theNMR^2^ terazosin-PGK1 structure with crystallographic PGK1 structures. (A) Overlay of the water-refined NMR2 structure (light pink, PDB ^32^PI) with the X-ray structure of the terazosin–hPGK1 complex (blue, PDB 4O33). Methyl-bearing residues used as intermolecular distance restraints in the NMR^2^ structure calculation are shown as sticks and labeled (A214, T254, L256, M311, L313, V341). (B) Overlay of the same NMR^2^ structure (light pink) with the nucleotide-bound, open-conformation X-ray structure of hPGK1 (red, PDB 2XE7), illustrating that the quinazoline ring of terazosin partially overlaps the adenine ring of ADP, while the two ligands occupy overall distinct volumes within the nucleotide-binding region.

The residues forming the hydrophobic pocket for the quinazoline and piperazine rings (F243, F291, F347, L256, M311, L313, V341) are consistent with previous findings, confirming that the quinazoline ring of terazosin occupies the same space as the ADP pyrimidine ring (Figure 4B), as proposed by Chen et al.^13^

In summary, the NMR^2^ structure establishes the solution-state binding mode of terazosin to hPGK1 and directly addresses whether the apparent activation behavior could arise from an alternative binding site. The structure converges on the same binding mode observed in the X-ray structure of the terazosin complex (4O33), which itself partially overlaps the canonical nucleotide-binding pocket, with no evidence for a distinct, well-defined secondary binding site that would have been strongly populated under solution conditions. These findings rule out alternative binding modes as the primary origin of terazosin-mediated activation and constrain mechanistic interpretations to modulation of conformational equilibria of PGK1.

Beyond its mechanistic implications, this study demonstrates that the NMR^2^ approach, in combination with selective unlabeling and partial methyl assignments, enables structure determination of large protein–ligand complexes under solution conditions even when distance restraints are sparse. This capability is particularly valuable when crystallographic data would be unavailable or when solution-state validation of binding modes is required.

## Conclusion

In this study, we used solution-state NMR to elucidate how terazosin modulates the activity of human phosphoglycerate kinase 1 (hPGK1). [^1^H–^15^N]-HSQC titration experiments demonstrate that terazosin binds competitively at a site that partially overlaps the nucleotide-binding pocket and shifts the conformational equilibrium of PGK1 toward open states, counteracting nucleotide-induced domain closure. These observations are consistent with a mechanism in which terazosin facilitates product release at low concentrations, while acting as a competitive inhibitor at higher concentrations.

To exclude alternative binding modes as the origin of this behavior, we determined the solution structure of the hPGK1-terazosin complex using the NMR^2^ approach. The resulting structure converges on a site partially overlapping with the canonical nucleotide-binding pocket and agrees closely with the crystallographic model, providing no evidence for a distinct secondary binding site under solution conditions. Together, these findings establish that terazosin’s functional effects arise from modulation of conformational equilibria within the canonical active site rather than from binding at an alternative site.

More broadly, this work illustrates how combining conformational NMR titration experiments with solution-state structure determination can resolve mechanistic ambiguities in enzyme modulation and clarify seemingly paradoxical ligand behavior.

## Material and Methods

### PGK1 Expression and Purification

PGK1 was expressed and purified as previously described.^13^ hPGK1 was recombinantly expressed using a hPGK1-PET28a vector in BL21 star *Escherichia coli* cells. The cells were grown in minimal M9 medium (^15^N or ^15^N,^13^C) at 37 °C until an OD of 0.8 was reached, the temperature was reduced to 25 °C, and expression was induced with 0.5 mM IPTG. In the case of selective unlabeling, 1 g/L of leucine, isoleucine, or threonine, or 0.5 g/L of alanine was added to the culture before induction.^24^ The cells were harvested after 16 h, resuspended in a buffer containing 20 mM sodium phosphate, 500 mM NaCl, 5 mM imidazole, and 5% glycerol at pH 7.9, microfluidized, and centrifuged, and the supernatant was loaded onto a nickel affinity column. The protein was eluted with 500 mM imidazole, dialyzed against an imidazole-free buffer, and the His-tag was cleaved with TEV. After reverse nickel affinity chromatography, the buffer was exchanged to NMR buffer containing 20 mM Tris, 100 mM NaCl, and 2 mM TCEP pH 7.4 using a PD10 desalting column.

### NMR Titration Experiments

All [^15^N,^1^H] titration experiments were acquired at 298 K on a Bruker Avance III HD 600 MHz spectrometer equipped with a cryoprobe, using TROSY or HSQC pulse sequences as specified below.

A concentration series of 0, 100, 200, 400, 800, and 2000 μM ADP was titrated to 250 μM apo hPGK1 in the presence of 10 mM MgCl2. The TROSY spectra were acquired with 16 scans, 220 (*t*_1, max_ (^15^N) = 45.1 ms) × 2048 (*t*_2, max_ (^1^H) = 125 ms) complex points, and an interscan delay of 0.8 s.

To form the transition state analog of hPGK1, a titration series with 0, 50, 100, 200, 400, 800, and 2000 μM ADP to 250 μM hPGK1 in the presence of 10 mM MgCl_2_, 30 mM NH_4_F, and 10 mM 3PG was conducted.

Terazosin was titrated with 0, 10, 100, 220, 470, and 1000 μM to 500 μM PGK1 in the presence of 10 mM MgCl_2_, 30 mM NH_4_F, and 10 mM 3PG. TROSY spectra with the same settings, but 8 scans were acquired.

The TSA opening of hPGK1 by terazosin was shown with a TROSY titration series of 0, 10, 30, 45, 60, 100, 125, 175, 250, 325, 400, 470, 566, 650, and 750 μM terazosin to 500 μM PGK1 in the presence of 10 mM MgCl_2_, 30 mM NH_4_F, 10 mM 3PG, and 1 mM ADP. The spectra were acquired with the same conditions as described above and 8 scans.

Additionally, terazosin (0, 10, 25, 50, 100, 250, and 1000 μM), in the absence or presence of 10 mM 3PG and MgF_3_^–^, and 3PG (0, 0.1, 0.25, 0.5, 1, 2, 4.15, and 10 mM) were titrated to 150 μM apo hPGK1. [^15^N,^1^H]-HSQC spectra were acquired with 64 scans, 200 (*t*_1_,max (^15^N) = 41.1 ms) × 2048 (*t*_2_,max (^1^H) = 122 ms) complex points, and an interscan delay of 0.89 s.

### 2D Filtered NOESY and [^13^C,^1^H]-HSQC

The F_1_-[^13^C,^15^N]-Filtered [^1^H,^1^H]-NOESY spectra were acquired with 800 μM ^13^C,^15^N-labeled hPGK1, 1 mM terazosin and 1 mM 3PG on a Bruker Avance 900 MHz ^1^H frequency spectrometer equipped with a cryoprobe with 400 (*t*_1,max_ (^1^H) = 17 ms) × 4096 (*t*_2,max_ (^1^H) = 152 ms) complex points, an interscan delay of 1.2 s, 196 scans and mixing times of 20, 60, 80, 100, and 120 ms.

Constant time [^13^C,^1^H]-HSQC spectra of 630 μM hPGK1 in the presence and absence of 700 μM terazosin were acquired on a Bruker AVANCE III 700 MHz ^1^H frequency equipped with a cryoprobe. The same spectra of either leucine, isoleucine, threonine, or alanine unlabeled ^13^C,^15^N-labeled hPGK1 were acquired.

### Data Analysis, NMR^2^ Structure Calculation and HADDOCK Water Refinement

#### NMR^2^

Spectra processing was performed in topspin 4.0.6, analysis and peak picking in CCPNMR 3.2.1,^25^ distance calculation with R software as previously shown by Pokharna et al.^23^ The NMR^2^ structure calculation was carried out with the intra and semiambiguous intermolecular distance restraints listed in Tables S1 and S2 using the NMR^2^ software. Thereby, PDB 4O33 was used as starting structure.^20^ The best structure calculated by the NMR^2^ software had a target function of 4.51 and van der Waals violation of 0.00003. The best resulting structure was submitted into HADDOCK 2.4 web portal water refinement tool.^26^

#### Titrations

The direct titrations in the fast chemical exchange were fitted with

1 $$\Delta \delta =\delta_{\max}\frac{K_{D}+P_{tot}+L_{tot}-\sqrt{({K_{D}+P_{tot}+L_{tot})}^{2}-4P_{tot}L_{tot}}}{2P_{tot}}$$

With

2 $$\Delta \delta_{obs}=\delta_{obs}-\delta_{free}$$

δ_free_ is the chemical shift of the unbound state, δ_obs_ is the observed chemical shift during the titration, *P*_tot_ is the total protein concentration, *L*_tot_ is the total ligand concentration, and *K*_D_ is the dissociation constant. Thereby, the geometrical mean of ^1^H and ^15^N resonances was calculated with

3 $$\Delta \delta_{obs}=\sqrt{\frac{{{\Delta \delta }_{obs,H}}^{2}+{{(0.14\times \Delta \delta }_{obs,N})}^{2}}{2}}$$

The slow chemical exchange was fitted with

4 $$\frac{I_{closed}}{I_{closed}+I_{open}}=\frac{K_{clo\sin g}+P_{tot}+L_{tot}-\sqrt{({K_{clo\sin g}+P_{tot}+L_{tot})}^{2}-4P_{tot}L_{tot}}}{2P_{tot}}$$

with *I* being the peak intensity of the open or closed state, respectively.

The competition titration to show the opening of PGK1 was fitted with

5 $$1-\frac{I_{closed}}{I_{closed}+I_{open}}=\frac{K_{opening,app}+P_{tot}+L_{tot}-\sqrt{({K_{opening,app}+P_{tot}+L_{tot})}^{2}-4P_{tot}L_{tot}}}{2P_{tot}}$$

With *K*_opening, app_ is the apparent opening equilibrium constant in the presence of ADP.

## Supplementary Material



## Notes

[^1^H,^15^N]-HSQC spectra of apo and ligand-bound hPGK1; chemical shift perturbation (CSP) maps and representative titration snapshots for ADP, terazosin, and 3-phosphoglycerate binding; equilibrium dissociation constants for ADP, terazosin, domain closing, and domain opening; HSQC snapshots of ADP- and terazosin-induced domain closure and reopening; CSPs under transition-state analogue conditions; time-dependent intramolecular and intermolecular NOE build-up curves and distance restraint tables for the hPGK1–terazosin complex; selective-unlabeling ct-[^13^C,^1^H]-HSQC spectra for partial methyl group assignment; methyl group assignments from NMR^2^ structure calculation (Figures S1–S10) and (Tables S1–S3) (PDF)

## References

[1] Watson H. C., Walker N. P., Shaw P. J., Bryant T. N., Wendell P. L., Fothergill L. A., Perkins R. E., Conroy S. C., Dobson M. J., Tuite M. F. (1982). Sequence and Structure of Yeast Phosphoglycerate Kinase. EMBO J..

[2] Bernstein B. E., Michels P. A. M., Hol W. G. J. (1997). Synergistic
Effects of Substrate-Induced
Conformational Changes in Phosphoglycerate Kinase Activation. Nature.

[3] Flachner B., Kovári Z., Varga A., Gugolya Z., Vonderviszt F., Náray-Szabó G., Vas M. (2004). Role of Phosphate Chain
Mobility of MgATP in Completing the 3-Phosphoglycerate Kinase Catalytic
Site: Binding, Kinetic, and Crystallographic Studies with ATP and
MgATP. Biochemistry.

[4] Pellegrini E., Juyoux P., von Velsen J., Baxter N. J., Dannatt H. R. W., Jin Y., Cliff M. J., Waltho J. P., Bowler M. W. (2024). Metal Fluorides—Multi-Functional
Tools for the Study of Phosphoryl Transfer Enzymes, a Practical Guide. Structure.

[5] Cliff M. J., Bowler M. W., Varga A., Marston J. P., Szabó J., Hounslow A. M., Baxter N. J., Blackburn G. M., Vas M., Waltho J. P. (2010). Transition State Analogue Structures of Human Phosphoglycerate
Kinase Establish the Importance of Charge Balance in Catalysis. J. Am. Chem. Soc..

[6] Zerrad L., Merli A., Schröder G. F., Varga A., Gráczer É., Pernot P., Round A., Vas M., Bowler M. W. (2011). A Spring-Loaded
Release Mechanism Regulates Domain Movement and Catalysis in Phosphoglycerate
Kinase*. J. Biol. Chem..

[7] Zhang K., Sun L., Kang Y. (2023). Regulation
of Phosphoglycerate Kinase 1 and Its Critical
Role in Cancer. Cell Commun. Signaling.

[8] He Y., Luo Y., Zhang D., Wang X., Zhang P., Li H., Ejaz S., Liang S. (2019). PGK1-Mediated Cancer Progression
and Drug Resistance. Am. J. Cancer Res..

[9] Fu Q., Yu Z. (2020). Phosphoglycerate Kinase
1 (PGK1) in Cancer: A Promising Target for
Diagnosis and Therapy. Life Sci..

[10] Siddique A. H. H., Kale P. P. (2024). Importance of Glucose
and Its Metabolism in Neurodegenerative
Disorder, as Well as the Combination of Multiple Therapeutic Strategies
Targeting α-Synuclein and Neuroprotection in the Treatment of
Parkinson’s Disease. Rev. Neurol..

[11] Chen H., Li Y., Gao J., Cheng Q., Liu L., Cai R. (2023). Activation
of Pgk1 Results in Reduced Protein Aggregation in Diverse Neurodegenerative
Conditions. Mol. Neurobiol..

[12] Kokotos A. C., Antoniazzi A. M., Unda S. R., Ko M. S., Park D., Eliezer D., Kaplitt M. G., De Camilli P., Ryan T. A. (2024). Phosphoglycerate
Kinase Is a Central Leverage Point
in Parkinson’s Disease–Driven Neuronal Metabolic Deficits. Sci. Adv..

[13] Chen X., Zhao C., Li X., Wang T., Li Y., Cao C., Ding Y., Dong M., Finci L., Wang J., Li X., Liu L. (2015). Terazosin Activates Pgk1 and Hsp90 to Promote Stress
Resistance. Nat. Chem. Biol..

[14] Chaytow H., Carroll E., Gordon D., Huang Y.-T., van der
Hoorn D., Smith H. L., Becker T., Becker C. G., Faller K. M. E., Talbot K., Gillingwater T. H. (2022). Targeting
Phosphoglycerate Kinase 1 with Terazosin Improves Motor Neuron Phenotypes
in Multiple Models of Amyotrophic Lateral Sclerosis. eBioMedicine.

[15] Cai R., Zhang Y., Simmering J. E., Schultz J. L., Li Y., Fernandez-Carasa I., Consiglio A., Raya A., Polgreen P. M., Narayanan N. S., Yuan Y., Chen Z., Su W., Han Y., Zhao C., Gao L., Ji X., Welsh M. J., Liu L. (2019). Enhancing Glycolysis Attenuates Parkinson’s Disease Progression
in Models and Clinical Databases. J. Clin. Invest..

[16] Riley M. J., Mitchell C. C., Ernst S. E., Taylor E. B., Welsh M. J. (2024). A Model
for Stimulation of Enzyme Activity by a Competitive Inhibitor Based
on the Interaction of Terazosin and Phosphoglycerate Kinase 1. Proc. Natl. Acad. Sci. U.S.A..

[17] Wang Y., Qian S., Zhao F., Wang Y., Li J. (2022). Terazosin
Analogs Targeting Pgk1 as Neuroprotective Agents: Design, Synthesis,
and Evaluation. Front. Chem..

[18] Marston J. P., Cliff M. J., Reed M. A. C., Blackburn G. M., Hounslow A. M., Craven C. J., Waltho J. P. (2010). Structural Tightening
and Interdomain Communication in the Catalytic Cycle of Phosphoglycerate
Kinase. J. Mol. Biol..

[19] Yagi H., Kasai T., Rioual E., Ikeya T., Kigawa T. (2021). Molecular
Mechanism of Glycolytic Flux Control Intrinsic to Human Phosphoglycerate
Kinase. Proc. Natl. Acad. Sci. U.S.A..

[20] Orts J., Wälti M. A., Marsh M., Vera L., Gossert A. D., Güntert P., Riek R. (2016). NMR-Based Determination of the 3D
Structure of the Ligand–Protein Interaction Site without Protein
Resonance Assignment. J. Am. Chem. Soc..

[21] Graham D. L., Lowe P. N., Grime G. W., Marsh M., Rittinger K., Smerdon S. J., Gamblin S. J., Eccleston J. F. (2002). MgF3–
as a Transition State Analog of Phosphoryl Transfer. Chem. Biol..

[22] Serimbetov Z., Baxter N. J., Cliff M. J., Waltho J. P. (2017). 1H, 15N,
13C Backbone
Resonance Assignments of Human Phosphoglycerate Kinase in a Transition
State Analogue Complex with ADP, 3-Phosphoglycerate and Magnesium
Trifluoride. Biomol. NMR Assign..

[23] Pokharna A., Torres F., Kadavath H., Orts J., Riek R. (2022). An Improved,
Time-Efficient Approach to Extract Accurate Distance Restraints for *N* MR ^2^ Structure Calculation. Magn. Reson..

[24] Lacabanne D., Meier B. H., Böckmann A. (2018). Selective Labeling and Unlabeling
Strategies in Protein Solid-State NMR Spectroscopy. J. Biomol. NMR.

[25] Skinner S. P., Fogh R. H., Boucher W., Ragan T. J., Mureddu L. G., Vuister G. W. (2016). CcpNMR AnalysisAssign:
A Flexible Platform for Integrated
NMR Analysis. J. Biomol. NMR.

[26] van
Zundert G. C. P., Rodrigues J. P. G. L.
M., Trellet M., Schmitz C., Kastritis P. L., Karaca E., Melquiond A. S. J., van Dijk M., de Vries S. J., Bonvin A. M. J. J. (2016). The HADDOCK2.2
Web Server: User-Friendly Integrative Modeling of Biomolecular Complexes. J. Mol. Biol..

