# Supplementary material for: Terazosin Activates or Inhibits Human Phosphoglycerate Kinase 1 on a Dose-Dependent Basis: Mechanistic and Structural Insights by NMR
Source: JACS Au. 2026 Sep 24;6(10):5623–30. doi: 10.1021/jacsau.6c00823 (PMC13714833; doi:10.1021/jacsau.6c00823)
Supplement: Supplementary file 1 [file au6c00823_si_001.pdf]

Terazosin Activates or Inhibits Human Phosphoglycerate Kinase 1 On a Dose-Dependent Basis: Mechanistic and Structural Insights by NMR

Matthias Bütikofer[a, c], Felix Torres[a, b], Dhiman Ghosh[a], Nils Lorz[a], Roland Riek*[a] and Julien Orts*[c]

[a] Institute for Molecular Physical Science
Vladimir Prelog Weg 2, 8093, Zürich, Switzerland
E-mail: riekr@ethz.ch
[b] Dr. F. Torres
NexMR AG
Wiesenstrasse 10A, 8952, Schlieren, Switzerland
[c] Department of Pharmaceutical Sciences, University of Vienna
Josef-Holaubek-Platz 2, A-1090 Vienna, Austria
E-mail: julien.orts@univie.ac.at

*Correspondence to:
roland.riek@phys.chem.ethz.ch; julien.orts@univie.ac.at

Supporting Information

KD ADP
KD TZ
KD Closing
KD Opening
Build Ups
Distance restraints

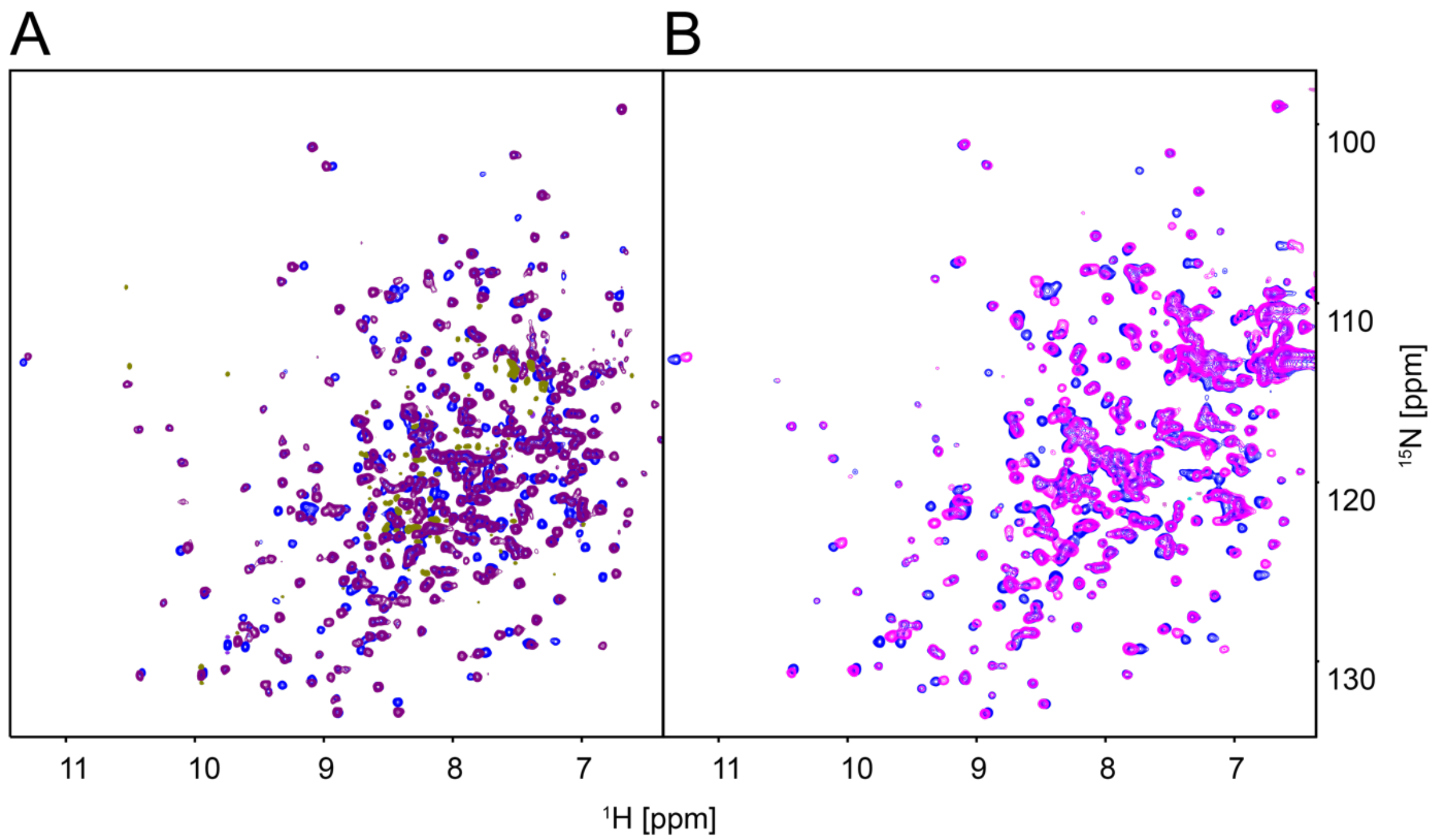

Figure S1: [^{15}N,^{1}H]-HSQC spectra of 200 µM apo PGK1 (blue) and bound to **(A)** 2000 µM ADP in the presence of 10 mM $MgCl_2$ (purple, neg. contour in olive) and **(B)** bound to 400 µM terazosin (pink). The spectra demonstrate chemical shift perturbations upon ligand binding, but the overall spectral fingerprint remains unchanged.

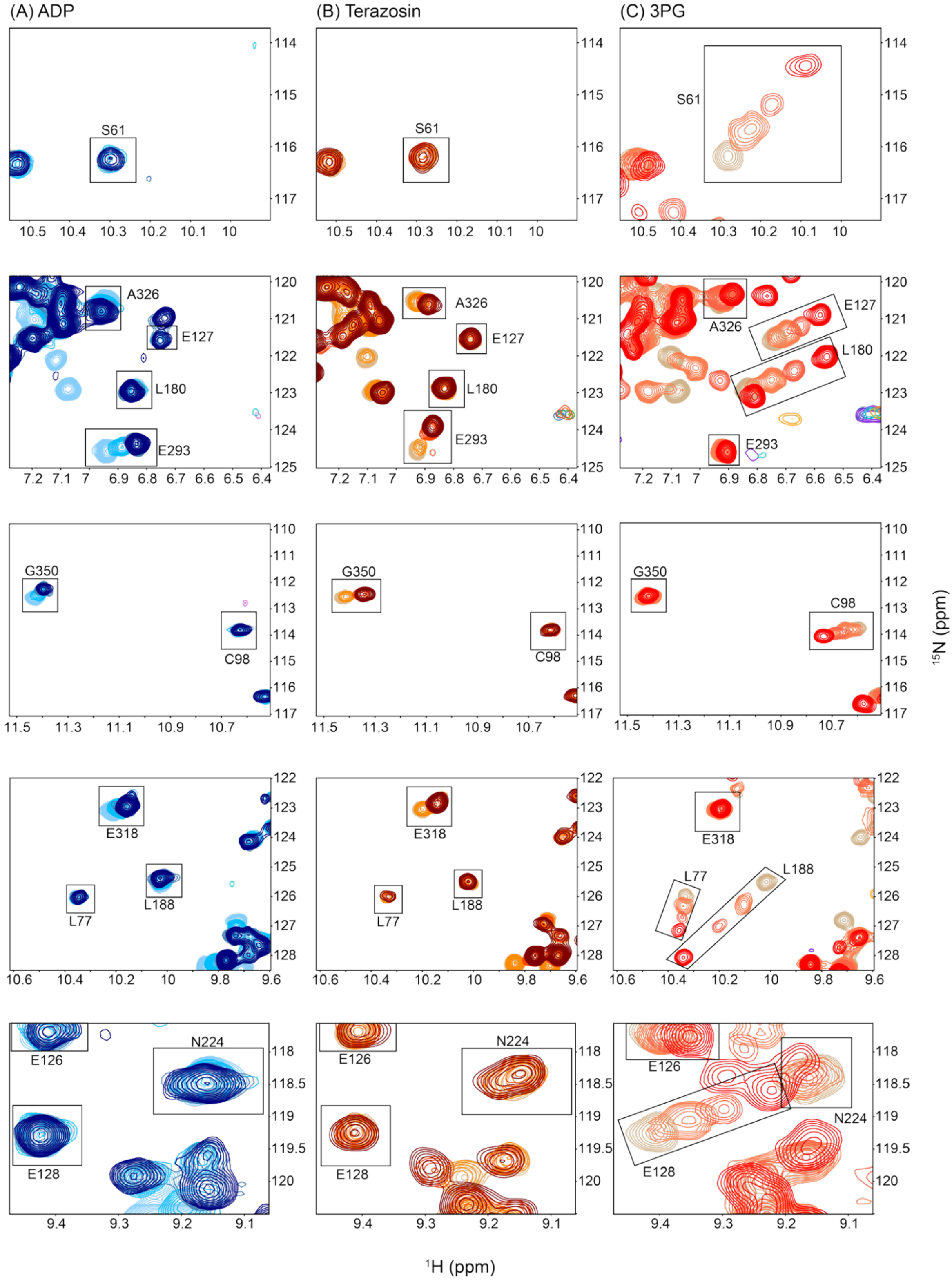

Figure S2. HSQC snapshots illustrating chemical shift perturbations upon ADP, terazosin, and 3PG binding. Zoomed [^{1}H, ^{15}N]-HSQC regions for ten representative residues, spanning both the N-terminal (S61, C98, L77, E126, E127, E128, L180, L188) and C-terminal (E293, A326, G350, E318) domains, shown for **(A)** ADP (0, 200, 800, 2000 μM, with 10 mM $MgCl_2$, light blue to navy), **(B)** terazosin (0, 25, 100, 1000 μM, tan to maroon), and **(C)** 3PG (0, 100, 250, 10000 μM; tan to red) titrated into apo PGK1. Residues are grouped into strips sharing a common ^{15}N chemical shift range.

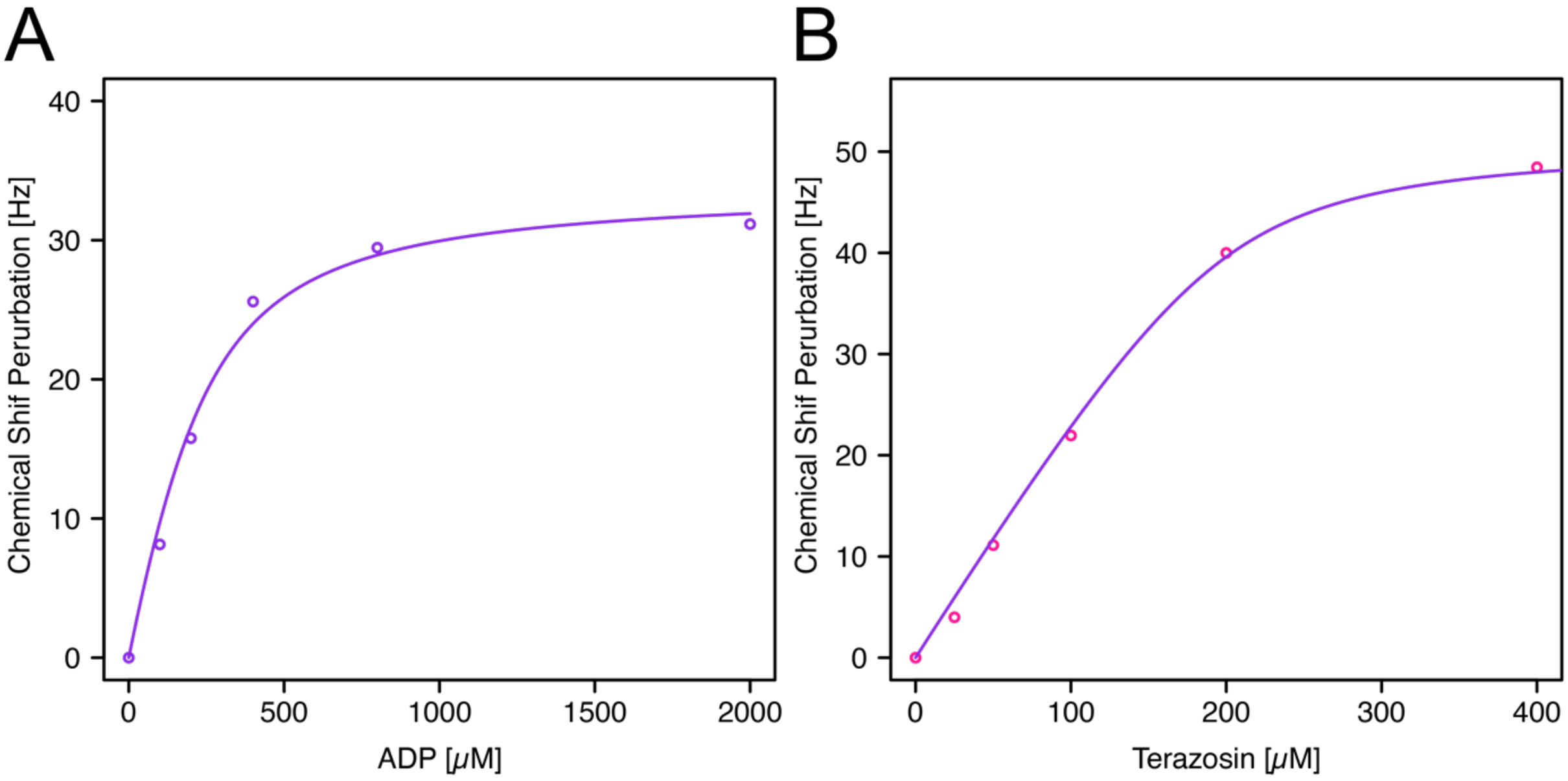

Figure S3: [^{15}N,^{1}H]-Chemical shift perturbations of PGK1 residue E318 with **(A)** ADP and **(B)** terazosin, corresponding to the titration series in Figure S1. The calculated affinities are 105 ± 25 μM for ADP and 13 ± 7 μM for terazosin.

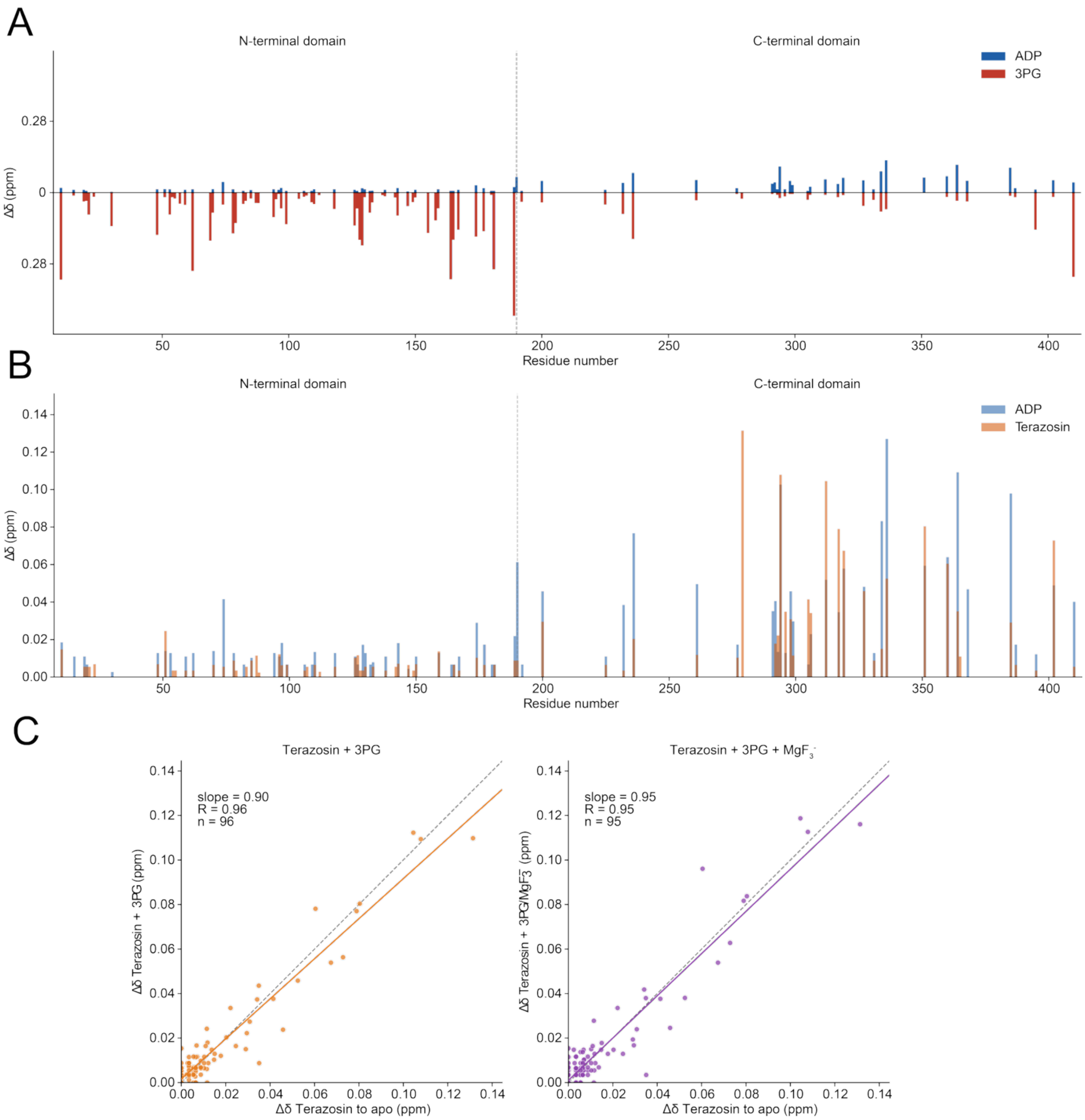

Figure S4: Chemical shift perturbation (CSP) mapping of terazosin, ADP and 3PG binding and counter-ligand independence. CSPs were calculated as according to Eq. 3 between the unbound and fully bound endpoints of each titration series, using assigned backbone amide resonances. The gray dashed line marks the boundary between the N-terminal (3PG-binding) and C-terminal (nucleotide-binding) domains. **(A)** Combined CSP map for ADP (blue, upward, 1 mM) and 3PG (red, downward, 10 mM) binding to apo PGK1, plotted against residue number. **(B)** CSP map comparing ADP (blue, 1 mM) and terazosin (orange, 1 mM) binding to apo PGK1, referenced to the same apo state. **(C)** Correlation plots comparing terazosin-induced CSPs in the presence of 3PG (left, orange, 10 mM) or 3PG/MgF_3^- (right, purple, 10 mM each) against terazosin-induced CSPs in apo PGK1. The dashed line indicates identity (y = x); the solid line is a linear fit to the data.

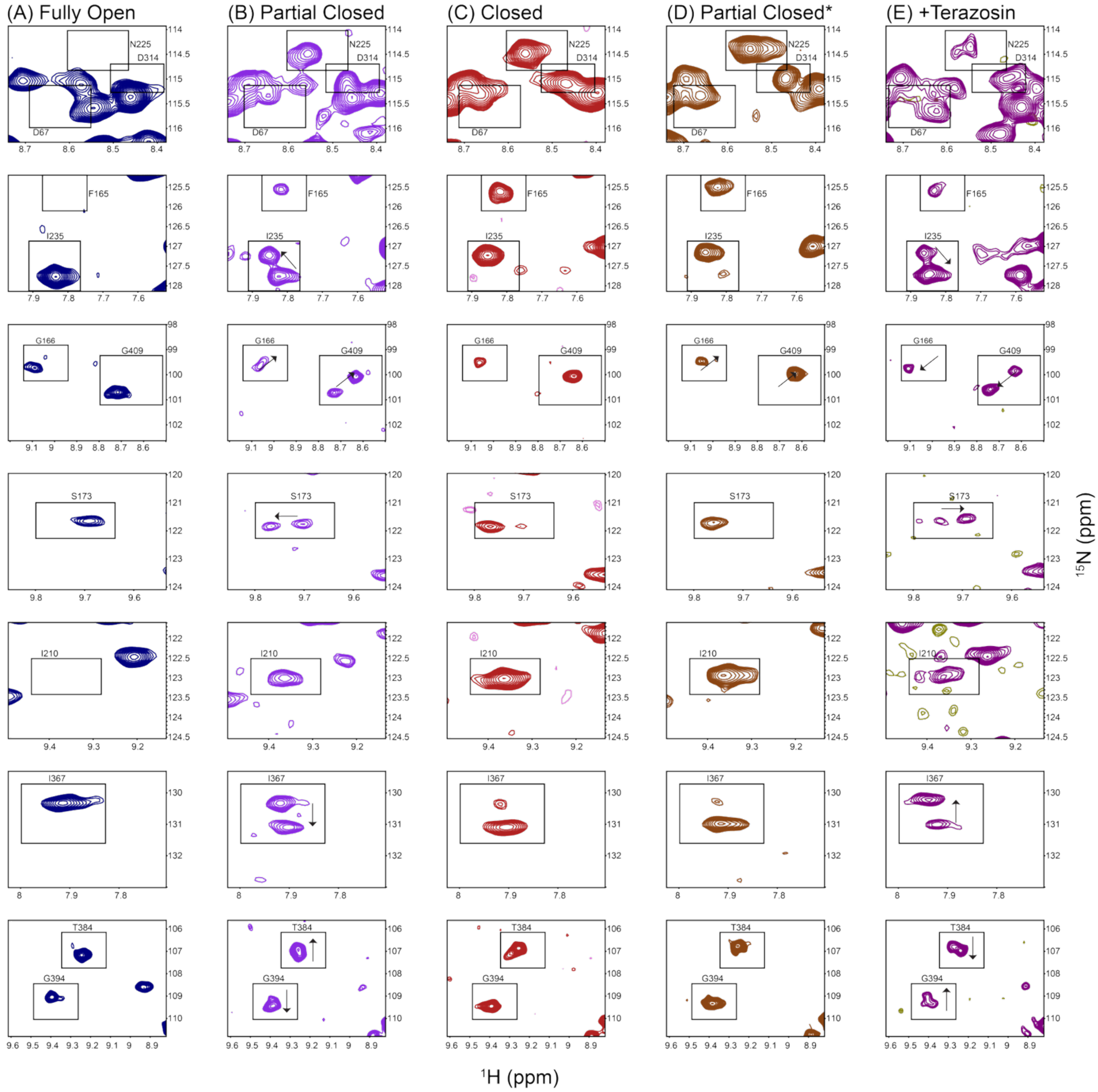

Figure S5. $[^{15}N,^{1}H]$-HSQC snapshots illustrating ADP- and terazosin-induced domain closure and reopening. Zoomed $[^{15}N,^{1}H]$-HSQC regions for 11 representative residues (D67, S173, I210, N225, I235, F165, I367, T384, G394, G409, and D314), spanning the N- and C-terminal domains, across five conditions: **(A)** 10 mM 3PG, 10 mM MgF_3^- (open), **(B)** + 200 μM ADP (partially closed), **(C)** + 2 mM ADP (closed), **(D)** +1 mM ADP, 0 μM terazosin (closed*), **(E)** +1 mM ADP, 650 μM terazosin. *(D) is an independently prepared closed reference for the terazosin titration shown in (E), at a lower ADP concentration (1 mM) than the closed state shown in (C) (2 mM ADP). Chemical shift positions and peak intensities were assigned based on the closed state deposited in BMRB 27022; the corresponding open-state peaks in (A) could not consistently be independently reassigned due to line broadening during the conformational exchange.

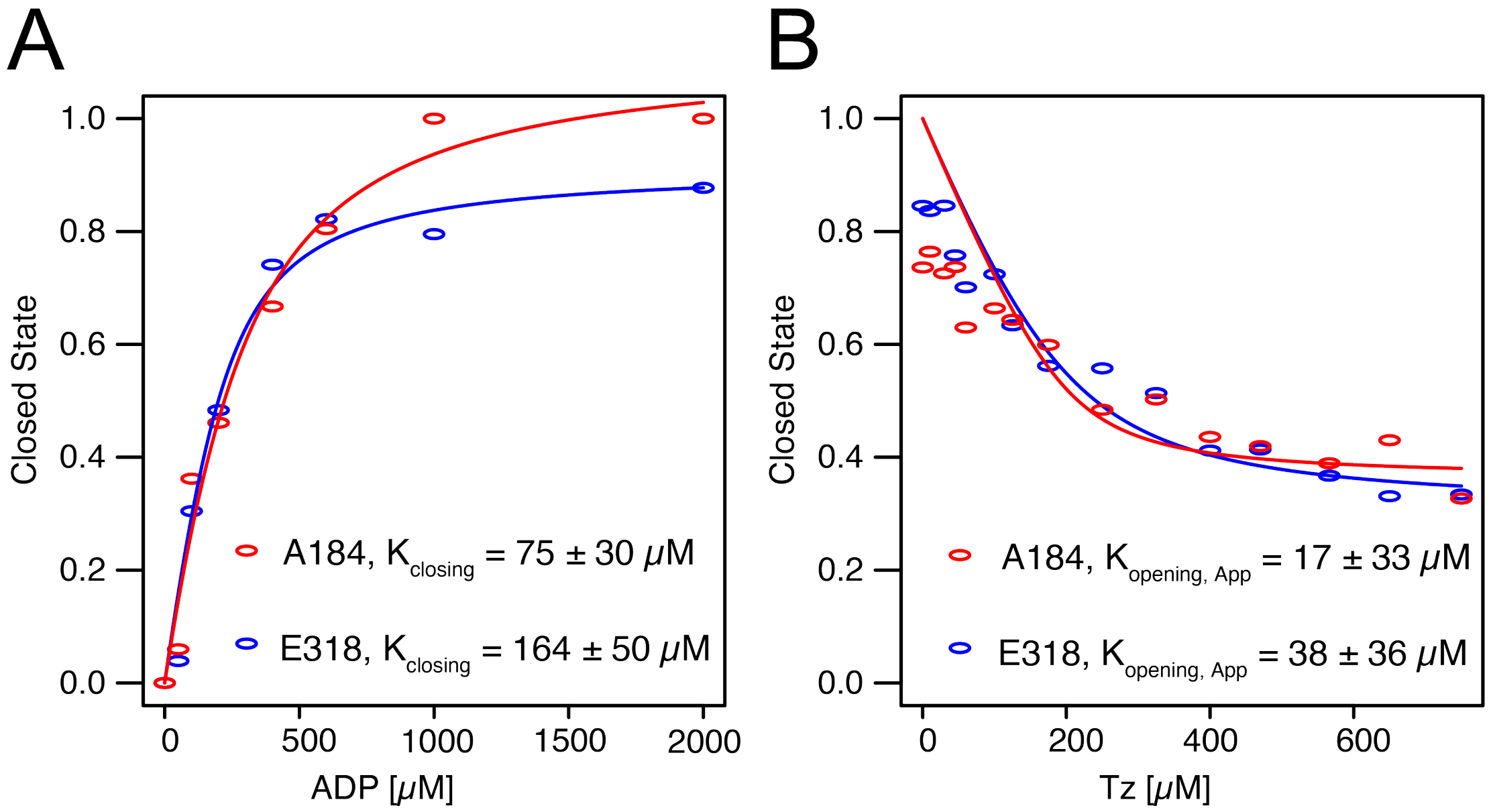

Figure S6: [^{15}N,^{1}H]-Chemical shift perturbation of PGK1 residues during titrations under TSA conditions (10 mM 3PG, 10 mM $MgCl_2$, and 30 mM NH_4F) with **(A)** ADP **(B**) terazosin in the presence of 10 mM ADP. The closing equilibrium constants ($K_{closing}$) and the apparent opening equilibrium constants ($K_{opening, App}$) for A184 (red) and E318 (blue) are displayed in the Figure. Note that these values represent apparent, conditional affinities measured under saturating counter-ligand conditions and are intended as demonstrations of relative affinity rather than a full thermodynamic linked-equilibrium analysis.

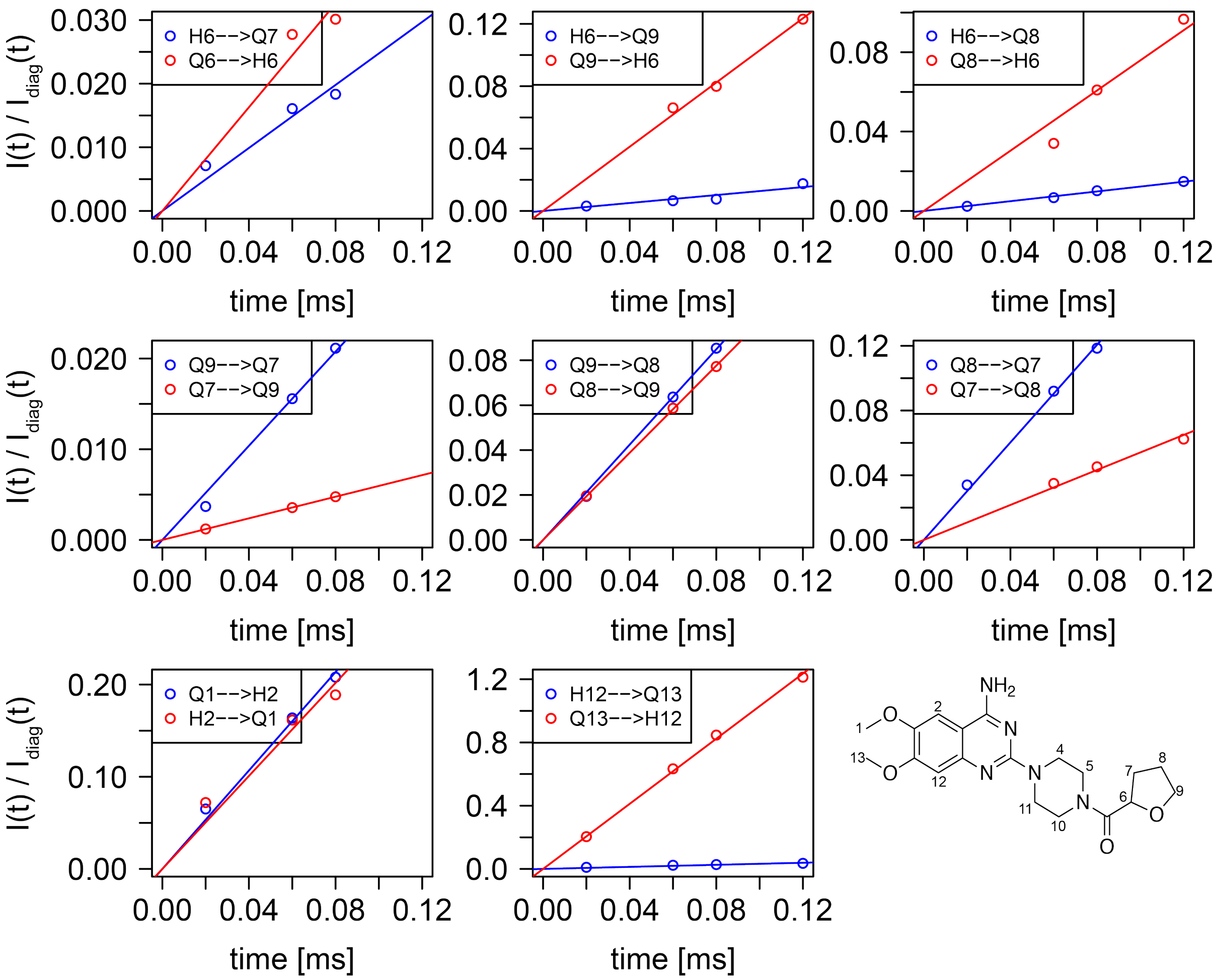

Figure S7: Time-dependent build-up curves of intramolecular NOEs within terazosin bound to hPGK1. Bidirectional NOE build-ups are fitted with linear regression (intercept = 0) for the extraction of NOE build-up rates used for distance conversion.[1]

Table S1: Intramolecular distance restraints of terazosin bound to hPGK1. The NOE buildup rates (σ_{ij}) extracted from Figure S7 were converted to distances by normalizing the median distance to 4 Å.[2]

F1-Assignment	F2-Assignment	Geometrical mean σ_{ij} [Hz]	r_{ij} [Å]
H6	Q7	0.22	4.2
H6	Q9	0.26	4.3
H6	Q8	0.22	4.3
Q9	Q7	0.06	5.3
Q9	Q8	0.51	3.7
Q8	Q7	0.45	3.8
Q1	H2	1.50	3.1
H12	Q13	1.10	3.3

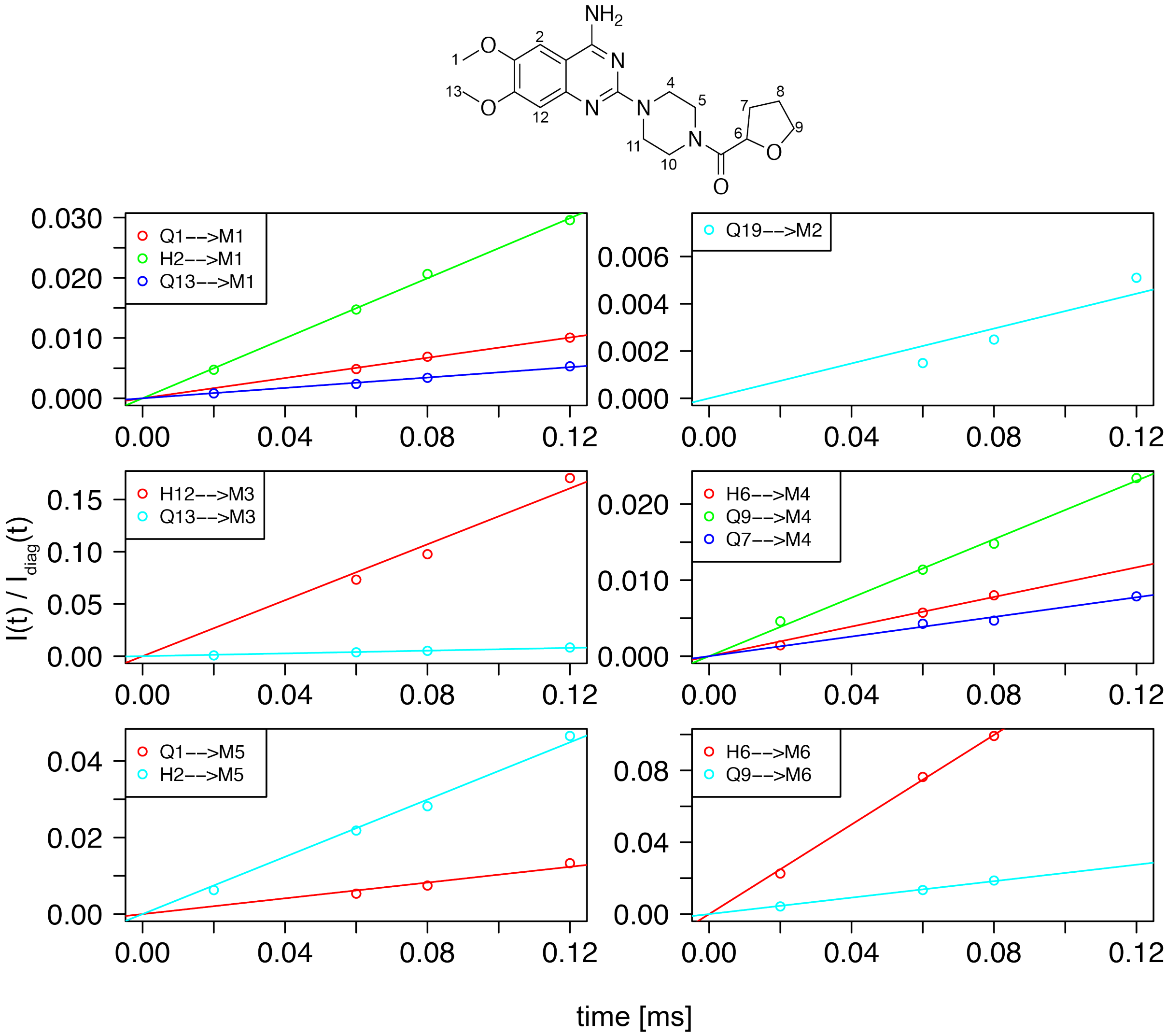

Figure S8: Time-dependent build-up curves of intermolecular NOEs between terazosin and hPGK1. Unidirectional NOE build-ups are fitted with linear regression (intercept = 0) to extract NOE build-up rates for distance calculations.[1]

Table S2: Intermolecular distance restraints between terazosin and unassigned methyl groups of hPGK1, derived from NOE build-up rates (σ_{ij}) in Figure S8 and were converted to distances by normalizing the median distance to 4.5 Å.[2]

F1-Assignment	F2-Assignment	σ_{ij} [Hz]	r_{ij} [Å]
M1 – Val	Q1	0.08	4.6
M1 – Val	H2	0.25	3.8
M1 – Val	Q13	0.04	5.1
M2 - Leu	H2	0.06	4.9
M3	H12	1.34	2.9
M3	Q13	0.07	4.8
M4 – Thr, Ala	Q9	0.10	4.5
M4 – Thr, Ala	Q8	0.19	4.0
M4 – Thr, Ala	Q7	0.06	4.8
M5 – Ala	Q1	0.10	4.4
M5 – Ala	H2	0.37	3.6
M6 - Met	H6	1.25	2.9
M6 - Met	Q8	0.22	3.9

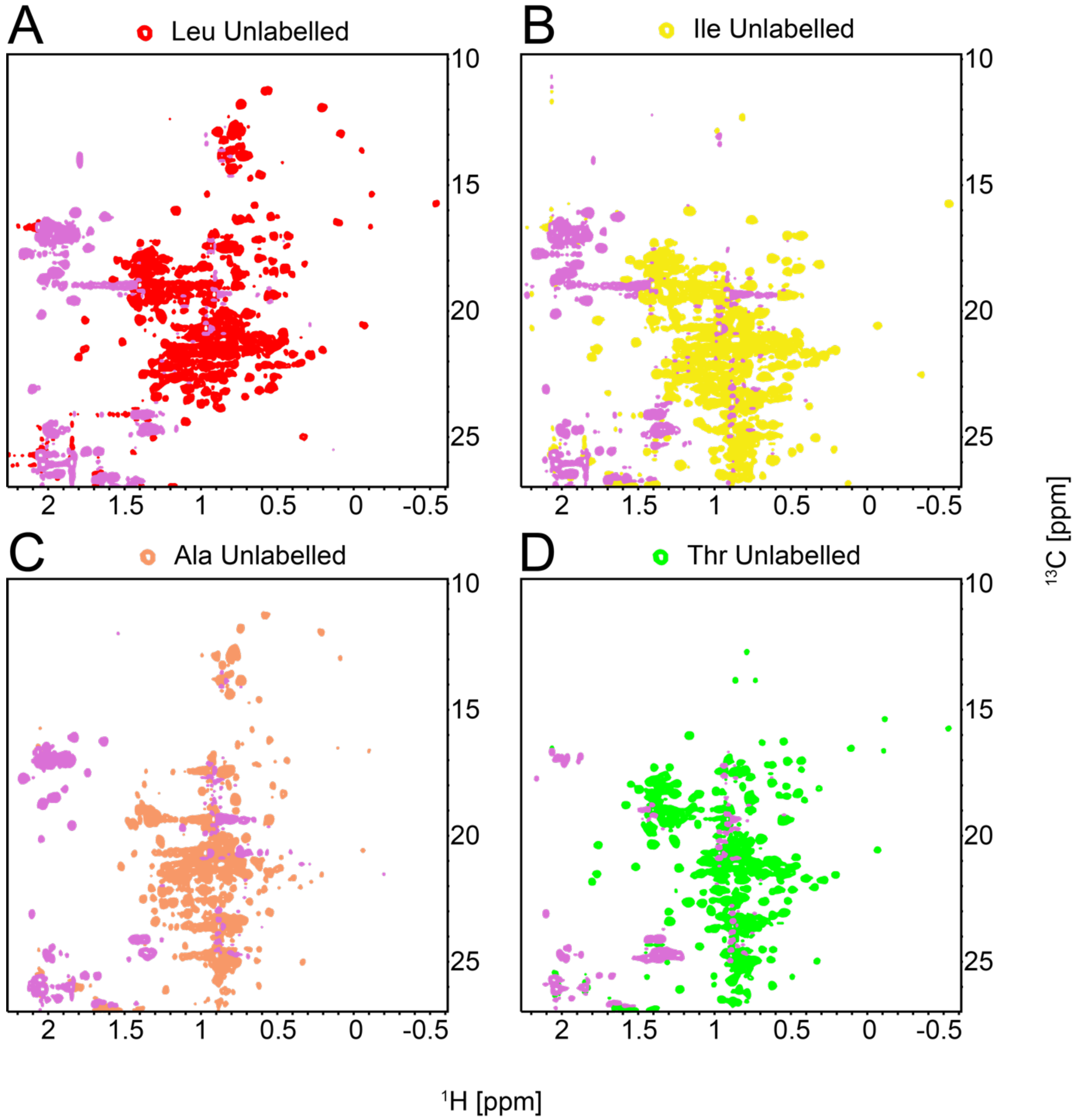

Figure S9: ct-[^{13}C, ^{1}H]-HSQC of uniformly labeled hPGK1 with selective unlabeling of methyl group-containing amino acids according to Lacabanne et al.[3] Spectra of samples with (A) leucine, (B) isoleucine, (C) alanine, or (D) threonine unlabeled enable partial methyl group assignment. Negative intensities are indicated with pink color.

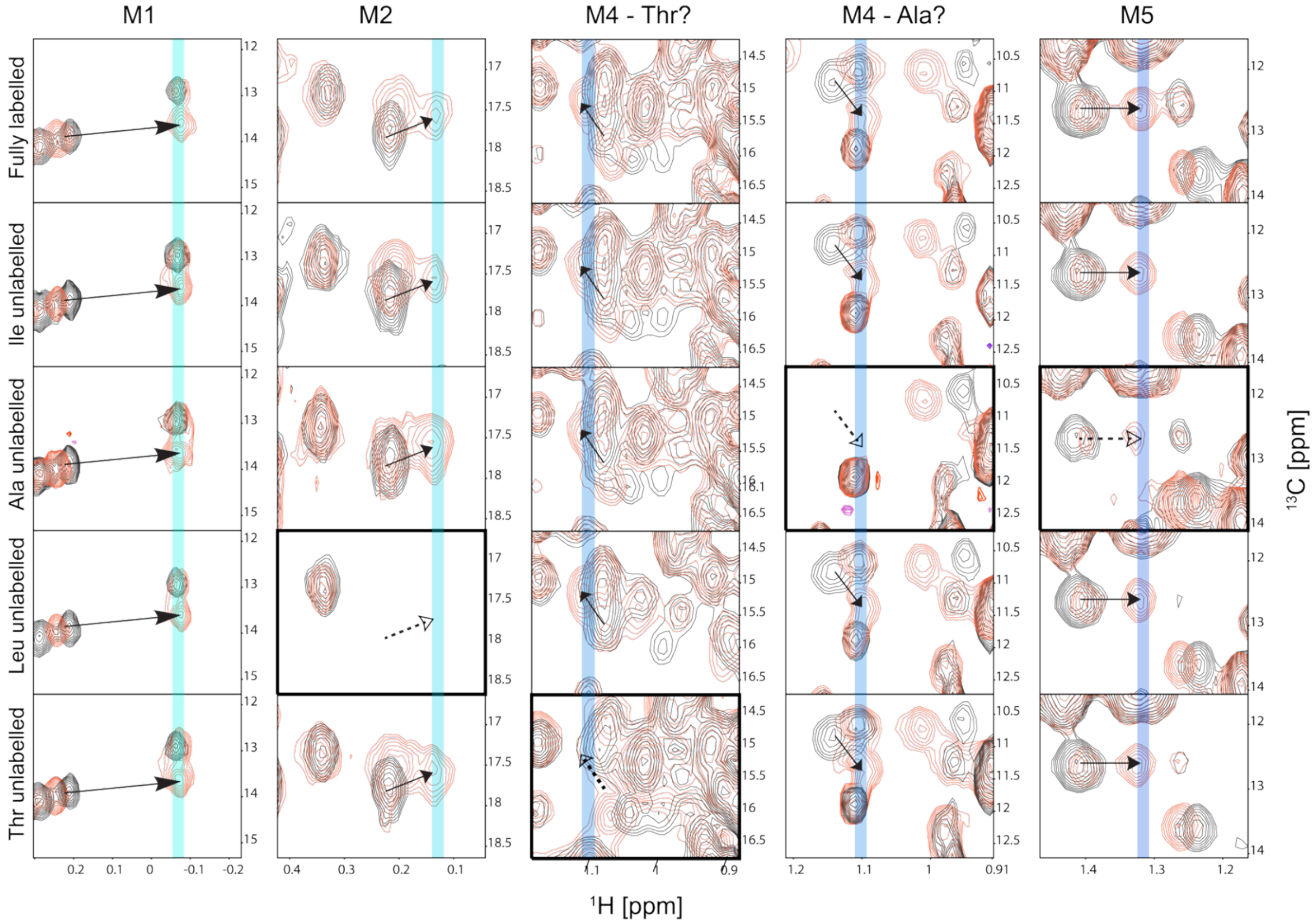

Figure S10: Zoomed [^{13}C, ^{1}H]-HSQC spectra of uniformly labeled hPGK1 with selective unlabeling of methyl-group-containing amino acids. Zoomed regions of [^{13}C, ^{1}H]-HSQC spectra illustrating methyl resonances involved in intermolecular NOEs with terazosin. The top row shows spectra of fully ^{13}C,^{15}N-labeled hPGK1, highlighting methyl chemical shift perturbations upon terazosin binding. The subsequent rows display overlays of spectra acquired from samples with selective unlabeled isoleucine, alanine, leucine, or threonine residues, as described by Lacabanne et al.[3] All spectra are acquired in the absence (black) or presence of 1 mM terazosin (red). Comparison of these spectra enables partial assignment of methyl resonances. Panels in which the selective unlabeling supports residue-type assignments are indicated by a thick border. Solid arrows track the chemical shift perturbation of a methyl resonance from its apo to its terazosin-bound position and dashed arrows with open arrowheads indicate the expected position of a resonance rendered undetectable in the corresponding unlabeled sample, consistent with loss of the underlying residue type. Colored vertical bars correspond to the intermolecular NOEs highlighted with the same colors in Figure 3A.

Table S3: Assignment based on the *N*MR^2 structure calculation of the methyl groups of hPGK1 involved in intermolecular NOEs to terazosin.

Methyl	Assignment
M1 - Val	Val 341 QG2
M2 – Leu	Leu 256 QD1
M3	Leu 313 QD2
M4 – Thr, Ala	Thr 254 QG2
M5 – Ala	Ala 214 QB
M6 – Met	Met 311 QE

(1) Pokharna, A.; Torres, F.; Kadavath, H.; Orts, J.; Riek, R. An Improved, Time-Efficient Approach to Extract Accurate Distance Restraints for *N* MR [2] Structure Calculation. *Magn. Reson.* **2022**, *3* (2), 137–144. https://doi.org/10.5194/mr-3-137-2022.

(2) Orts, J.; Wälti, M. A.; Marsh, M.; Vera, L.; Gossert, A. D.; Güntert, P.; Riek, R. NMR-Based Determination of the 3D Structure of the Ligand–Protein Interaction Site without Protein Resonance Assignment. *J. Am. Chem. Soc.* **2016**, *138* (13), 4393–4400. https://doi.org/10.1021/jacs.5b12391.

(3) Lacabanne, D.; Meier, B. H.; Böckmann, A. Selective Labeling and Unlabeling Strategies in Protein Solid-State NMR Spectroscopy. *J Biomol NMR* **2018**, *71* (3), 141–150. https://doi.org/10.1007/s10858-017-0156-z.
